# Anaphylactic reactions to pomegranate: identification and characterization of eliciting IgE-reactive components

**DOI:** 10.1186/2045-7022-1-S1-P88

**Published:** 2011-08-12

**Authors:** Arnd Petersen, Andreas Kleinheinz, Uta Jappe

**Affiliations:** 1 Research Center Borstel, Clinical and Molecular Allergology, Borstel, Germany; 2 Dermatological Center Buxtehude, Buxtehude, Germany

## Background

Reports on anaphylactic reactions to exotic fruits, e.g. kiwi and dragon fruit, are increasing. Therefore, the allergenic risk assessment and the identification of allergenic components are important. 9 cases of allergic reactions to pomegranate have already been reported, but the responsible allergens have so far not been characterized in detail.

## Patients and methods

A 26 year old female patient developed erythema, swelling of the ears and pruritus within 10 min after ingestion of pomegranate. She is allergic to birch pollen and apple. Prick and IgE tests revealed a positive reaction to mites, tree and grass pollen, ambrosia, mugwort and apple. Scratch test was positive for pomegranate, the seeds showing a stronger reaction than the juice. Oral provocation test with kiwi, peach and cherry was negative. The serum was analysed for IgE reactivity by Western blotting. As extracts served different sections of pomegranate (juice, extract and seed). IgE-reactive protein bands were analyzed and identified by protein sequencing and homology screening.

## Results

The IgE-binding patterns differed between juice with one band of 9 kDa and the seeds with three IgE-reactive components of about 21, 16 and 6 kDa. The 9 kDa allergen was identified as a lipid transfer protein (LTP), which revealed a 77% sequence identity to the LTP from peach. While the 21 kDa protein of the seeds was N-terminally blocked, the 16 kDa component showed sequence similarity to Bet v 1-homologous proteins, which is in line with the patient’s reactivity to birch and apple. The 6 kDa protein revealed no significant sequence similarities to proteins in the databases. Tryptic mass fingerprinting is in progress to identify the allergens in more detail.

## Conclusions

Pomegranate is a relevant allergen source which shows different IgE-reactive components in its compartments. The structural characterization of these components is necessary to define their allergenicity and the underlying IgE-binding epitopes for improving in vivo and in vitro diagnosis and to estimate the potency of novel allergens in the future.

